# Identification of *bZIP* Gene Family in *Bergenia purpurascens* and Functional Characterization of *BpbZIP37* Under Heat Stress

**DOI:** 10.3390/ijms262110262

**Published:** 2025-10-22

**Authors:** Qiankun Zhu, Wenqing Wang, Tianxiang Chen, Feiyang Yan, Jie Chen, Zongxiang Jiang, Nuomei Xu

**Affiliations:** Sichuan Engineering Research Center for Biomimetic Synthesis of Natural Drug, School of Life Science and Engineering, Southwest Jiaotong University, Chengdu 610031, China; wwqswjtu@yeah.net (W.W.); ctxswjtu@yeah.net (T.C.); yfyswjtu@yeah.net (F.Y.); cjswjtu@yeah.net (J.C.); jzxswjtu@yeah.net (Z.J.)

**Keywords:** *B. purpurascens*, *bZIP* gene family, heat stress

## Abstract

*Bergenia purpurascens*, a perennial herb with significant medicinal value, thrives in harsh high-altitude environments but faces threats from global warming-induced heat stress. Basic leucine zipper (bZIP) transcription factors play crucial roles in plant growth, development, and stress responses, yet their functions in *B. purpurascens* remain unstudied. In this study, we identified 55 *bZIP* genes (*BpbZIP1–55*) from *B. purpurascens* transcriptome data. Expression pattern analyses identified several tissue-specific expressed *BpbZIP* genes, as well as eight heat stress-induced *BpbZIP* genes. Among them, *BpbZIP37* was the most significantly induced by heat stress and was used for functional characterization via over-expression in *Arabidopsis thaliana.* The results indicated that *BpbZIP37* can enhance the heat tolerance of plants by reducing reactive oxygen species accumulation and increasing activities of antioxidant enzymes. This study provides new insights into exploring the functions of *bZIP* genes in *B. purpurascens*, highlighting *BpbZIP37* as an important regulator of heat stress responses, which lays a foundation for plant breeding improvement and resource conservation tailored to climate change.

## 1. Introduction

*Bergenia purpurascens* is a perennial herb of the genus Bergenia in the family Saxifrage, which is widely distributed at high altitudes in East Asia, northern South Asia and southeastern Central Asia [1,2]. Its growing environment is particularly special, mostly found in the understory, scrub-covered areas, open meadows and rock crevices between 2700 and 4800 m above sea level, which often have extreme and variable climatic conditions, demonstrating *B. purpurascens*’ excellent adaptability to harsh environments [1,2]. During the summer in the habitat of *B. purpurascens*, the daytime temperature can rise to 15–25 °C, while the nighttime temperature drops sharply, leading to a diurnal temperature range of 10–15 °C. However, due to the occurrence of extreme weather in recent years, the maximum summer temperature in some habitats has exceeded 25 °C. In winter, it can tolerate low temperatures ranging from −10 °C to −5 °C, and some high-altitude populations can even withstand lower temperatures for a short period [3].

The medicinal value of *B. purpurascens*, particularly the bergenin extracted from it, has been widely recognized as an effective treatment for chronic bronchitis [1,2,4]. Long-term excessive and disorderly mining has led to a decline in the natural renewal capacity of wild *B. purpurascens* [5,6]. With the increasingly serious global warming, some high-latitude or high-altitude areas have seen shortened cold seasons, the warm and hot season is extended, which constitutes a potential threat to the growth and development of *B. purpurascens* and its accumulation of medicinal ingredients [7]. Therefore, an in-depth study of the adaptation mechanism of *B. purpurascens* to high-temperature environments is of great significance for ensuring the sustainable use of *B. purpurascens* resources, optimising artificial cultivation strategies and developing new heat-resistant varieties.

Basic leucine zipper (bZIP) is a class of transcription factors widely found in eukaryotes [8]. All bZIPs share a common conserved structural domain containing 60–80 amino acids [8]. Its N-terminal is a basic region consisting of 18 basic amino acids, containing a nuclear localization signal (NLS) and an N-x7-R/K motif that binds to specific DNA sequences [9].The C-terminal is a leucine ZIP region with a unique amino acid composition that tends to form sparse surface interactions. It often forms homo- or heterodimers in the form of α-helices via hydrophobic surface interactions to mediate transcriptional activation or repression.

Plant bZIP transcription factors play critical roles in plant growth, development, and stress resistance [10]. *AtbZIP34* in *Arabidopsis thaliana* is required for pollen wall patterning and the control of several metabolic pathways in developing pollen [11]. *ZmbZIP17* in maize regulates the accumulation of storage substances, and *ZmbZIP4* over-expression increases the number of lateral roots [12]. *OsbZIP23*, *OsbZIP66* and *OsbZIP72* in rice interact with *OsMFT2* to positively regulate ABA-responsive gene expression, affecting seed germination [13]. Regarding stress resistance research, over-expression of *AtABP9* enhances the heat tolerance of transgenic plants [14]. *GmbZIP44*, *GmbZIP62* and *GmbZIP78* in Soybean improve salt and cold tolerance in transgenic *A. thaliana* [15]. Additionally, members of the *bZIP* gene family are involved in the regulation of secondary metabolite synthesis in various medicinal plants [16]. SmbZIP1 transcription factor in *Salvia miltiorrhiza* Bunge var. negatively regulates tanshinone biosynthesis [17]. *AabZIP19* in *Artemisia annua* regulates the biosynthesis of phenolic and flavonoid compounds through promoter interaction with *AaPAL1* [18]. *EsbZIP1*, *EsbZIP2* and *EsbZIP5* in *Eleutherococcus senticosus* negatively regulate the expression of saponin synthase genes and triterpenoid saponin synthesis [19].

Currently, there is no research on the bZIP gene family in *B. purpurascens*. Based on our previously obtained transcriptome sequencing data [1,2], 55 members of *B. purpurascens bZIP* (*BpbZIP*) gene family were identified and subjected to sequence analyses using bioinformatics methods, their expression patterns in different tissues and under heat stress were further investigated via RNA-seq and real-time quantitative PCR (RT-qPCR). Additionally, a heat stress-induced member, *BpbZIP37*, was selected for biological function investigation under heat stress. This study provides new insights into exploring the functions of *bZIP* genes in *B. purpurascens*, as well as plant breeding improvement and resource conservation tailored to climate change.

## 2. Results

### 2.1. Identification of CcAP2/ERFs of bZIP Proteins in B. purpurascens

Based on the Hidden Markov Model (HMM) profile of the conserved bZIP domain (PF00170) from the Pfam database, a total of 55 bZIP proteins were identified from the *B. purpurascens* transcriptome-derived protein sequences. These proteins were designated in order as BpbZIP1 to BpbZIP55. To further characterize these bZIP proteins, their physicochemical properties were analyzed. The 55 identified BpbZIP proteins exhibited substantial variation in sequence length, theoretical isoelectric point (pI), molecular weight, instability index, and aliphatic index (Table S1).

Specifically, the length of these proteins ranged from 137 to 802 amino acid residues, with predicted isoelectric points varying from 4.59 to 11.18 and an average pI of 7.45 (Table S2). The grand average of hydropathicity (GRAVY) values ranged from −1.207 to −0.376, indicating that all 55 BpbZIP proteins are hydrophilic. Subcellular localization predictions performed using Plant-mPLoc indicated that all BpbZIP proteins are localized in the nucleus, suggesting that they may function as transcription factors within the nuclear compartment.

### 2.2. Phylogenetic and Motif Analysis of BpbZIPs Proteins

**Figure 1:**
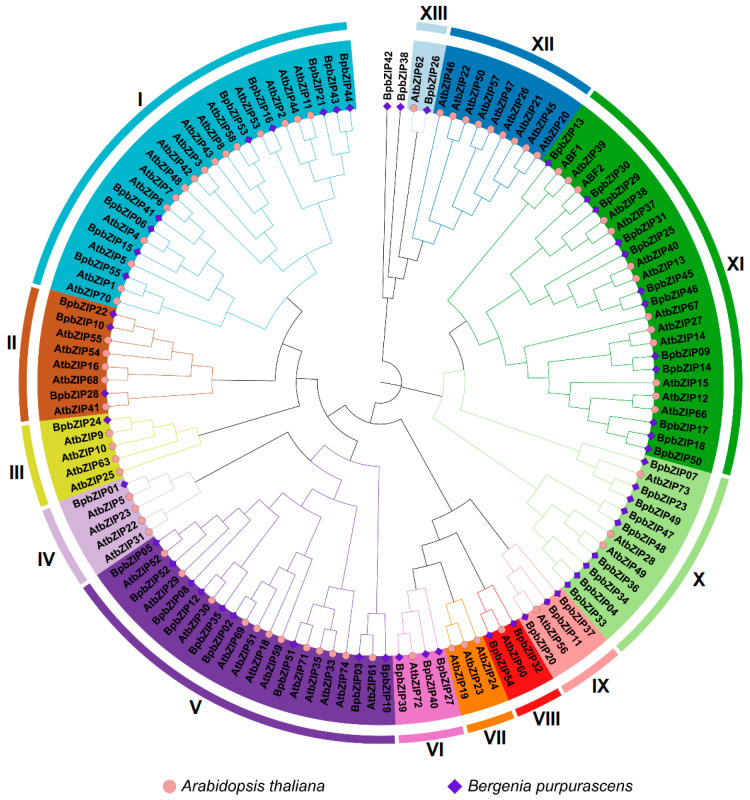
Phylogenetic tree of bZIP proteins from *B. purpurascens* and *A. thaliana*. Different subfamilies are shown in different colours. Roman numerals (I–XIII) represent subfamily numbers.

To classify the bZIP protein subfamilies in *B. purpurascens* and elucidate their evolutionary relationships with the *A. thaliana* bZIP proteins, a phylogenetic tree was constructed using 55 BpbZIP protein sequences from *B. purpurascens* and 78 bZIP protein sequences from *A. thaliana*. As shown in Figure 1, based on the established classification of the *A. thaliana* bZIP superfamily, the 55 BpbZIP proteins clustered into 13 subfamilies, with members grouped within the same subfamily likely sharing functional similarities. Specifically, as indicated, 12 BpbZIP proteins were assigned to subgroup XI; subgroups I, V, and X each contained 9 BpbZIP proteins, while other subgroups comprised fewer members. Notably, no BpbZIP proteins were grouped into subgroups VII and XII. Additionally, BpbZIP42 and BpbZIP38 did not cluster with any identified subfamily and thus formed separate branches independently.

**Figure 2:**
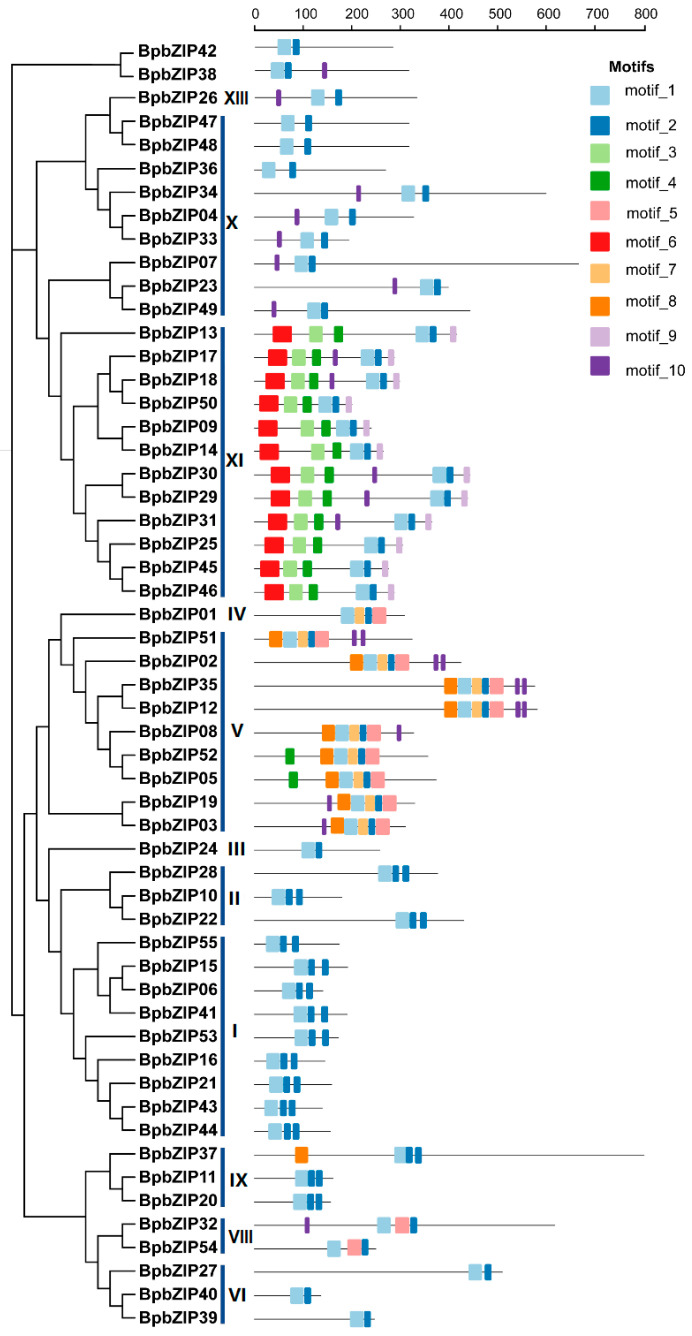
Conserved Motif Analysis of the bZIP Gene Family in *B. purpurascens*. Different motifs (motif 1–10) are indicated by different colours. Roman numerals (I–XIII) represent subfamily numbers.

Further analysis using the online MEME tool identified 10 conserved motifs within these proteins, revealing commonalities and variations in gene structure and functional domains within each subfamily. Members within the same subfamily exhibited similar gene structural distributions (Figure 2). Each bZIP structural domain consists of a basic DNA-binding region and a leucine zipper (ZIP) domain. The results indicated that all 55 BpbZIP proteins contained a highly conserved N-X7-R motif (motif 1) in their N-terminal regions, representing the DNA-binding domain and including a nuclear localization signal. Moreover, the motif composition displayed notable differences among various subfamilies. Specifically, subgroup XI proteins contained the highest number of predicted motifs, characterized by the exclusive presence of motif 6 at the N-terminal and motif 9 at the C-terminal regions. Subgroup V uniquely contained motif 8. This distinct motif distribution pattern among subfamilies likely indicates unique roles and underlying mechanisms for each bZIP subgroup in performing specific biological functions, thus providing insights into the potential evolutionary logic and molecular basis underlying the structural and functional diversity of the BpbZIP protein family.

### 2.3. Expression Pattern Analysis of BpbZIP Genes in Different Tissues of B. purpurascens

**Figure 3:**
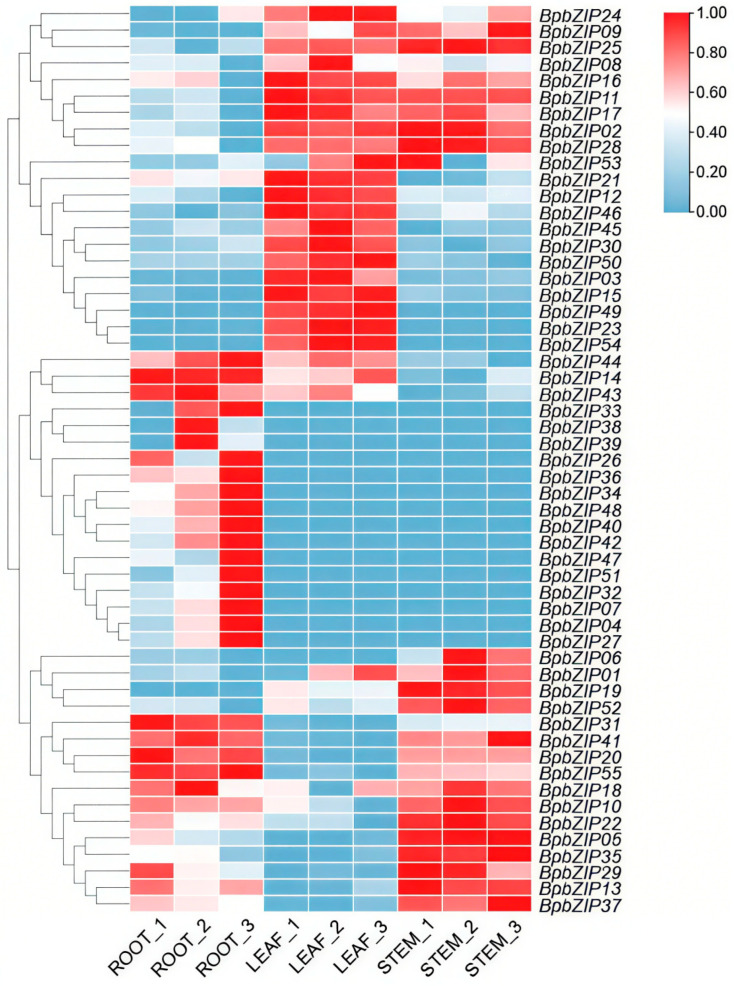
Expression pattern of *BpbZIP* genes in three different tissues. Each sample was repeated three times. Gene expression data were log2-transformed and normalized to the zero-to-one scale.

To investigate the expression patterns of the *BpbZIP* genes in various tissues (roots, stems, and leaves) of *B. purpurascens*, we conducted bidirectional clustering analyses of RNA-seq data derived from these tissues. The expression levels of *BpbZIP* genes were quantified using TPM (transcripts per million) values (Table S3), and visualized as heatmaps using TBtools-II to illustrate gene expression across different tissues (Figure 3). The expression heatmap revealed that the majority of *BpbZIP* genes exhibited variable expression patterns rather than constitutive expression. *BpbZIP03*/*12*/*15*/*21*/*23*/*30*/*45*/*46*/*49*/*50*/*54* were highly expressed in leaves, *BpbZIP04*/*07*/*26*/*27*/*31*/*32*/*33*/*34*/*36*/*38*/*39*/*40*/*42*/*47*/*48*/*51* were highly expressed specifically in roots, *BpbZIP06*/*19*/*52* were highly expressed specifically in stems, *BpbZIP01*/*02*/*09*/*11*/*16*/*17*/*24*/*25*/*28*/*53* were highly expressed in stems and leaves, *BpbZIP14*/*43*/*44* were highly expressed specifically in roots and leaves, while *BpbZIP06*/*10*/*13*/*18*/*20*/*22*/*29*/*35*/*37*/*41*/*55* were highly expressed specifically in roots and stems. These findings not only highlight the complexity of tissue-specific expression patterns within the *BpbZIP* gene family but also provide essential clues for further exploration of the functional roles these genes play in various biological processes related to the growth and development of *B. purpurascens*.

### 2.4. Expression Pattern Analysis of BpbZIP Genes Under Heat Stress

**Figure 4:**
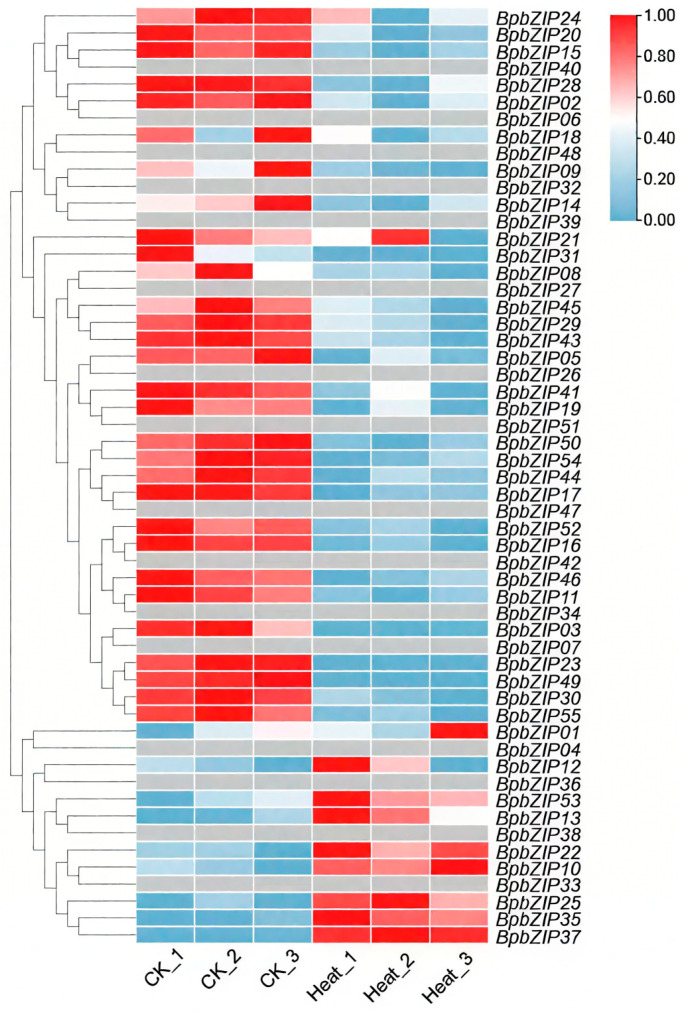
Expression patterns of *BpbZIP* genes under heat stress. Each sample was repeated three times. Gene expression data were log2-transformed and normalized to the zero-to-one scale.

**Figure 5:**
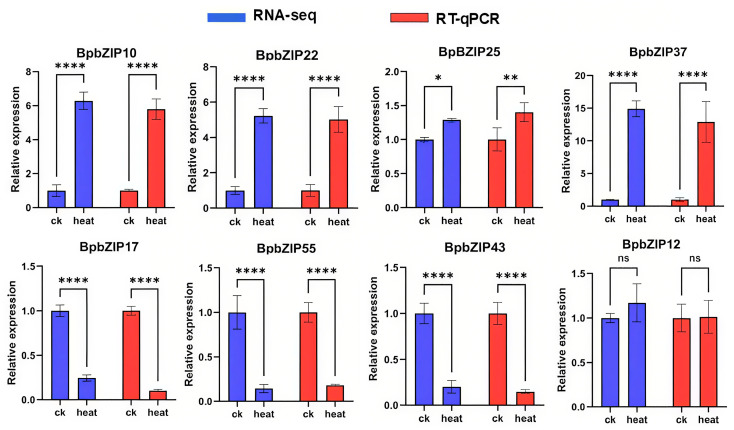
RT-qPCR analysis of the expression patterns of eight *BpbZIP* genes under heat stress and normal conditions. Statistical significance was determined using Student’s *t*-test (* *p* < 0.05, ** *p* < 0.01, **** *p* < 0.0001, “ns” represents “no significant difference”, *n* = 3).

RNA-seq analysis was conducted on the leaves of *B. purpurascens* under heat stress and control conditions to investigate the effects of heat treatment on *BpbZIP* gene expression. To visualize the changes in gene expression levels following heat treatment, a heatmap was generated (Figure 4) based on the TPM values (transcripts per million) of *BpbZIP* genes under both stress and control conditions (Table S4). A total of 32 *BpbZIP* genes exhibited differential expression. Among them, the expression of *BpbZIP10*/*12*/*13*/*22*/*25*/*35*/*37*/*53* was up-regulated by heat stress, with *BpbZIP37* exhibiting the largest fold change. These genes were identified as heat-responsive candidate genes and may serve as key targets for further investigating the molecular mechanisms and adaptive strategies of *B. purpurascens* in response to heat stress. To further validate the reliability of the RNA-seq results, eight *BpbZIP* genes (*BpbZIP10*/*12*/*17*/*22*/*25*/*37*/*55*/*43*) were selected for RT-qPCR analysis. The RT-qPCR results revealed that the expression patterns of these eight genes were consistent with those observed in the RNA-seq data (Figure 5), confirming the accuracy of the experimental results.

### 2.5. Transient Expression and Subcellular Localization Analysis of BpbZIP37

**Figure 6:**
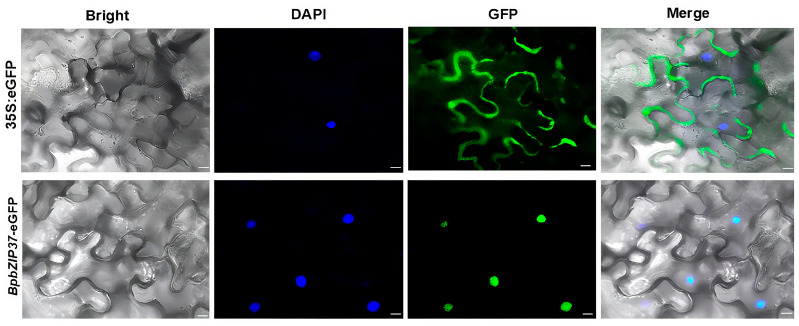
Microscopic examination of *BpbZIP37*-eGFP transient expression and subcellular localization. Bar = 20 μm. DAPI: 4′,6-Diamidino-2-phenylindole, a double-stranded DNA fluorescent dye for labeling cell nuclei. The blue and green fluorescence were emitted by DAPI and GFP respectively.

To investigate the biological function of *BpbZIP37*, a vector of *35S:BpbZIP37-eGFP-tNOS* was constructed and introduced into *N. benthamiana* by *Agrobacterium*-mediated transformation for transient expression and subcellular localization analysis of *BpbZIP37*. The results showed that *BpbZIP37* was successfully transiently expressed in *N. benthamiana,* and the expressed protein is mainly localized in the nucleus, which is consistent with the subcellular localization of most transcription factors (Figure 6).

### 2.6. Over-Expression of BpbZIP37 Enhanced Heat Tolerance in A. thaliana

**Figure 7:**
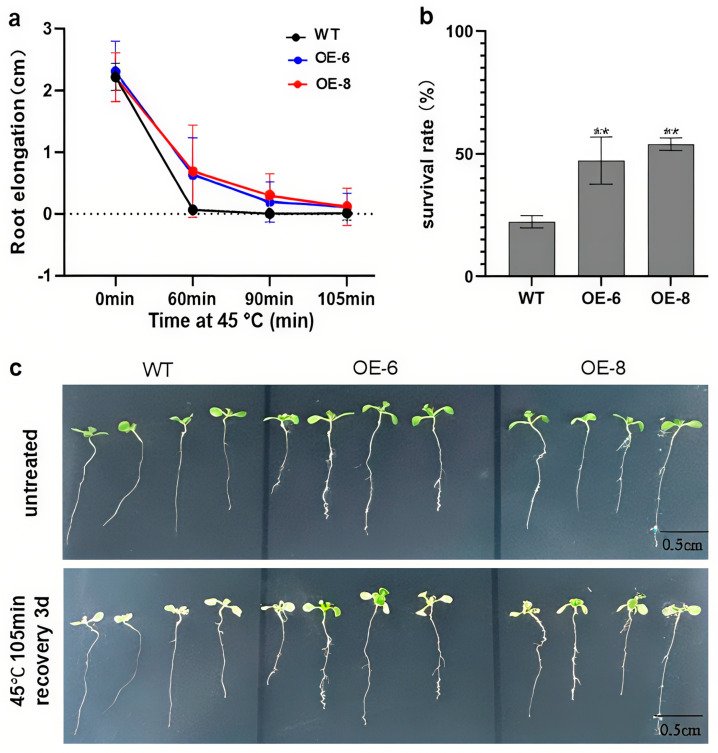
Effect of *BpbZIP37* over-expression on the heat tolerance of *A. thaliana* seedlings. (**a**) Root length statistics of 4-day-old seedlings after 45 °C heat treatment for different times and 5 days of recovery. (**b**) Survival rates of seedlings under heat stress. (**c**) Phenotypes of 4-day-old seedlings after heat stress at 45 °C for 105 min followed by 3 days of recovery. Statistical significance was assessed using Student’s *t*-test with WT as the control. (** *p* < 0.01, *n* = 3, Student’s *t*-test). Bar = 0.5 cm.

To further investigate the role of the *BpbZIP37* gene in heat stress response, the over-expression vector was introduced into the model plant *A. thaliana* using *Agrobacterium*-mediated transformation, and transgenic lines OE-6 and OE-8 exhibiting high expression levels (Figure S1) were selected for subsequent analyses. In the experiments, 7-day-old seedlings of wild type (WT), OE-6, and OE-8 were subjected to heat stress at 45 °C for durations of 60 min, 90 min, and 105 min, respectively, and then allowed to recover under normal conditions for 5 days. Root elongation was recorded and analyzed (Figure 7 and Figure S2). Results indicated that root growth in WT seedlings ceased after exposure to heat stress for only 60 min, whereas OE-6 and OE-8 seedlings continued to exhibit root growth even after prolonged heat stress (up to 90 min and 105 min), along with a significantly lower rate of leaf bleaching compared to WT.

To evaluate seedling survival rates, seven-day-old seedlings (OE-6, OE-8, and WT lines) were subjected to heat stress (45 °C, 105 min) and subsequently allowed to recover for 3 days under normal growth conditions (Figure 7). The results showed that the survival rate of WT seedlings was only 22.2%, whereas the two over-expression lines, OE-6 and OE-8, exhibited significantly higher survival rates ranging from 47.5% to 50%. Collectively, these findings suggest that the over-expression of *BpbZIP37* can effectively reduce the detrimental effects of heat stress on plant growth and development, thereby significantly enhancing plant thermotolerance.

### 2.7. Over-Expression of BpbZIP37 Reduced the Accumulation of ROS

**Figure 8:**
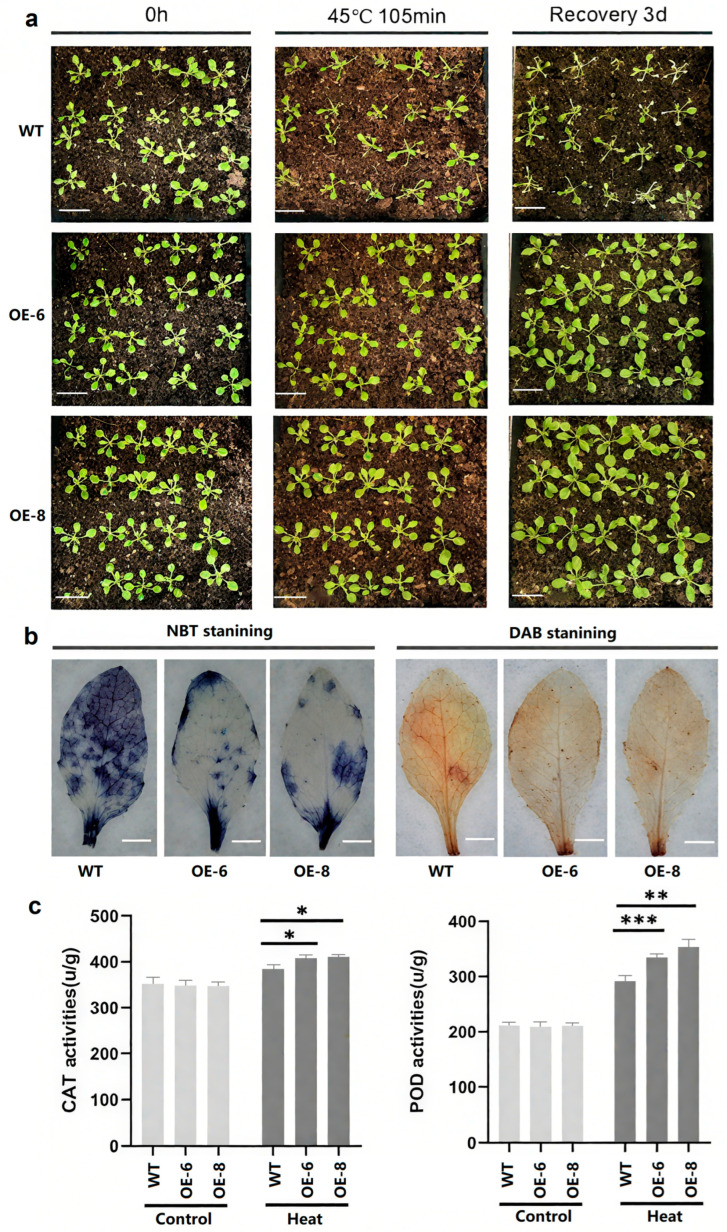
Detection of ROS levels and antioxidant enzyme activities in WT and *BpbZIP37-OE* plants after heat stress. (**a**) 18-day-old seedlings were treated with 45 °C heat stress for 105 min followed by 3 days of recovery. Bar = 3 cm. (**b**) NBT and DAB staining of leaves from WT, OE6, and OE8 plants following heat treatment at 45 °C for 105 min. Bar = 3 mm. (**c**) CAT and POD activity of WT and transgenic lines OE2 and OE3 under control and heat conditions. (* *p* < 0.05, ** *p* < 0.01, *** *p* < 0.001, *n* = 3, Student’s *t*-test).

Eighteen-day-old *A. thaliana* seedlings grown in soil were exposed to heat stress at 45 °C for 105 min, followed by a recovery period of 3 days. As shown in Figure 8a, wild-type seedlings exhibited obvious wilting and poor growth, whereas the *BpbZIP37* transgenic plants showed relatively minor changes.

ROS accumulation in rosette leaves from the same position of wild-type and transgenic *A. thaliana* plants was analyzed (Figure 8b). DAB staining results indicated that staining intensity in the two transgenic lines was lighter compared to the wild type. Similarly, NBT staining revealed a smaller blue-stained area in transgenic lines following heat treatment, suggesting stronger heat tolerance in these transgenic plants.

Quantifying POD and CAT enzyme activities provides insights into the plant antioxidant system’s responsiveness and helps elucidate mechanisms underlying tolerance to oxidative stress. Spectrophotometric analysis revealed that POD and CAT activities significantly increased in the transgenic lines OE-6 and OE-8 compared with wild type after heat treatment (Figure 8c). The contents of soluble proteins and proline in the OE-6 and OE-8 lines were significantly higher than those in the wild type (WT) (Figure S3). Thus, *BpbZIP37* can enhance plant antioxidant capacity by up-regulating POD and CAT activities, as well as increasing the contents of soluble proteins and proline, thereby further improving heat tolerance.

## 3. Discussion

### 3.1. B. purpurascens in the Face of Global Climate Change

As a high-altitude perennial herb with well-documented medicinal value, *B. purpurascens* has evolved remarkable adaptability to extreme alpine environments characterized by variable climates [5,6]. However, the wild resources of this plant now faces dual threats, long-term un-regulated harvesting, which has eroded its natural renewal capacity, and global warming, which shortens cold seasons and extends warm/hot periods in high-latitude/high-altitude regions-disrupting its growth and medicinal metabolite accumulation [5,6,7]. Climate change poses a unique risk to high-altitude medicinal plants, as their narrow ecological niches limit adaptive migration; thus, deciphering their stress adaptation mechanisms is critical for resource conservation and sustainable utilization [7].

This study addresses this gap by focusing on the bZIP transcription factors, a family widely conserved in eukaryotes and pivotal for plant growth, development, and abiotic stress responses [8,10]. Prior to this work, no research had explored *bZIP* genes in *B. purpurascens*, the identification of *BpbZIPs* and functional characterization of *BpbZIP37* fill a key void in understanding how this medicinally important species copes with heat stress, a primary climate change-induced threat.

### 3.2. Phylogenetic and Structural Insights on BpbZIP Gene Family

The study identified 55 bZIP genes from *B. purpurascens* transcriptome data, the number shows a certain difference from that of other plants. For comparison, 78 *bZIP* genes are present in *A. thaliana* [20], 89 in *Oryza sativa* [21], and 161 in *Glycine max* [22], which suggesting that *BpbZIP* family might be evolutionarily compact but functionally diverse. Phylogenetic analysis clustered 55 *BpbZIPs* into 13 subfamilies (with no members in subgroups VII and XII) using *A. thaliana bZIPs* as references, a grouping that aligns with the conserved evolutionary trajectory of the *bZIP* family [20]. Notably, subgroup XI contained the largest number (12) of *BpbZIPs*, while subgroups I, V, and X each had 9 members-implying functional specialization within subfamilies, as observed in other plants [20,23]. *BpbZIP38*/*42* did not cluster with any identified subfamily, which suggests that they may possess unique functions.

Conserved motif analysis further supported functional divergence, all 55 BpbZIPs contained the canonical N-x7-R/K motif (motif 1), a hallmark of the bZIP DNA-binding domain and nuclear localization signal (NLS) [20,23], confirming their identity as transcription factors. Subfamily-specific motifs (e.g., motif 6 in subgroup XI, motif 8 in subgroup V) suggest unique regulatory roles-for example, Subgroup XI bZIPs in *A. thaliana* are known to mediate abscisic acid (ABA) signaling and drought tolerance [20,23], raising the possibility that *B. purpurascens* subgroup XI BpbZIPs may contribute to multiple stress responses. This structural conservation, paired with subfamily-specific divergence, aligns with the “conserved core, specialized functions” model of bZIP evolution in plants [20], thereby providing clues for the study of the evolution and biological functions of BpbZIPs.

### 3.3. BpbZIP37 Might Be a Key Regulator of Heat Stress Response

Heat stress triggered differential expression in 32 *BpbZIP* genes, with eight of these genes showing up-regulation. Given its most significant induction by heat stress, *BpbZIP37* was identified as a primary candidate for functional validation. This aligns with studies in other plants, for example, *A. thaliana AtABP9* over-expression enhances heat tolerance [14], and *G. max GmbZIP44*/*62*/*78* improve salt and cold tolerance in transgenic *A. thaliana* [15]. The upregulation of *BpbZIP37* under heat stress thus supports a conserved role for bZIPs in mediating thermotolerance across angiosperms.

RT-qPCR validation confirmed the RNA-seq results, ensuring the reliability of *BpbZIP37* as a heat-responsive gene. Subcellular localization analysis further showed BpbZIP37 accumulates in the nucleus-consistent with its predicted role as a transcription factor, as typically function in the nucleus to regulate target gene expression [20,23]. This localization is critical, nuclear-localized bZIPs can bind to cis-elements (e.g., ACGT motifs) in the promoters of stress-responsive genes, triggering downstream defense pathways [20,23]. For BpbZIP37, nuclear localization suggests it may directly or indirectly regulate heat stress-related genes (e.g., antioxidant enzymes, chaperones) to enhance tolerance.

### 3.4. Mechanisms of BpbZIP37-Mediated Heat Tolerance

A key finding of this study is that *BpbZIP37* over-expression can improve the plant heat tolerance through the reduction in ROS accumulation and elevation of antioxidant enzyme activity, which are well-established mechanisms in plant stress biology [24]. This mechanism aligns with that maintaining ROS homeostasis via antioxidant enzymes is a core strategy for plant stress tolerance [25,26]. Heat stress induces excessive ROS production (e.g., H_2_O_2_, O_2_^−^), which damages membranes, proteins, and nucleic acids [25]. To counter this, plants activate antioxidant systems, including peroxidase and catalase-key enzymes that scavenge H_2_O_2_ and O_2_^−^ [26]. By linking BpbZIP37 to ROS scavenging, this study identified a concrete molecular pathway underlying the heat adaptation of *B. purpurascens,* which provided a target for genetic manipulation to improve plant thermotolerance.

The mechanism by which *BpbZIP37* regulates plant heat tolerance through the antioxidant pathway has also been reported in other plants. For example, BdiVIP1A, a bZIP member in *Brachypodium distachyon*, acts as a hub gene in heat stress response [27]. It significantly enhances heat tolerance in *B. distachyon* by activating the antioxidant enzyme system consisting of SOD, POD, and CAT to scavenge ROS. This indicates that bZIP transcription factors in *B. distachyon* enhance plant heat tolerance by regulating the antioxidant enzyme system. Heat shock proteins (HSPs) are essential in plant heat tolerance. Studies have revealed that maize *ZmbZIP60* connects the unfolded protein response (UPR) with the heat stress response (HSR) [28]. Mutants with *ZmbZIP60* knockout showed impaired HSR under high temperatures and prevented the upregulation of *HSP* genes under high temperatures. The expression of maize *heat shock transcription factor 13* (*ZmHSFTF13*) was impaired in *ZmbZIP60* mutants, and the *ZmHSFTF13* promoter is a target of ZmbZIP60 in maize protoplasts. These findings suggest that ZmbZIP60 functions in maize heat tolerance by regulating *HSFTF13* to influence the expression of *HSP* genes. Whether *BpbZIP37* regulates plant heat tolerance through HSF or HSP requires further investigation.

### 3.5. Practical Implications for Resource Conservation and Breeding

High-altitude plants often lack genetic diversity for rapid adaptation to climate change, making targeted genetic improvement essential [29]. These findings have direct applications for *B. purpurascens* resource management and plant breeding under climate change. First, *BpbZIP37* represents a candidate gene for developing heat-resistant *B. purpurascens* varieties via genetic engineering-critical for preserving this medicinal species as global warming intensifies. Second, the identification of tissue-specific and heat-responsive *BpbZIPs* provides markers for optimizing artificial cultivation. For example, root-enriched *BpbZIPs* could guide soil amendment strategies to enhance root growth, while heat-responsive genes like *BpbZIP37* could be used to screen heat-tolerant accessions in breeding programs.

### 3.6. Limitations and Future Directions

While this study makes significant contributions, it has limitations that warrant future investigation. First, functional validation was conducted exclusively in *A. thaliana*, a model plant distantly related to *B. purpurascens* (Saxifragaceae). To confirm the role of *BpbZIP37* in its native host, future studies should use CRISPR-Cas9 or RNA interference (RNAi) to knock down *BpbZIP37* in *B. purpurascens* and assess heat tolerance-an approach successfully applied to other medicinal plants [30,31,32].

Second, the study did not identify direct target genes of *BpbZIP37*. Chromatin immunoprecipitation sequencing (ChIP-seq) or yeast one-hybrid (Y1H) assays could reveal which heat stress-related genes BpbZIP37 regulates (e.g., antioxidant enzyme genes, heat shock proteins), clarifying its position in the heat stress regulatory network. Additionally, investigating interactions between BpbZIP37 and other TFs could uncover complex regulatory cascades, as bZIPs often function as dimers to mediate transcriptional responses [20,23]. In addition to *BpbZIP37*, this study also identified other members whose expression is induced by heat stress, such as *BpbZIP10*/*12*/*13*/*22*/*25*/*35*/*53*. However, whether these members regulate plant heat tolerance and the specific regulatory mechanisms involved remain unknown and require further investigation.

Finally, this study focuses exclusively on heat stress; it is important to note that *B. purpurascens* endures a suite of alpine stresses, including cold, drought, salinity, and ultraviolet (UV) radiation. Evaluating the role of *BpbZIP37* in other stresses would determine if it confers broad-spectrum tolerance—a trait highly valuable for breeding. Plant bZIPs have also been shown to be involved in the regulation of plant secondary metabolism [16,33]; therefore, whether BpbZIPs participate in the metabolic regulation of medicinal components in *B. purpurascens* warrants further attention.

## 4. Materials and Methods

### 4.1. Plant Materials and Growth Conditions

*B. purpurascens* plants collected from Wenchuan Botanical Garden, Sichuan Province, China, were transplanted into a soil-vermiculite mixture and cultivated at 22 °C under a 16 h light/8 h dark cycle. Its ITS2 and psbA-trnH sequences were identified using DNA barcoding technology [1,2], and combined with its morphological characteristics, it was confirmed to be *B. purpurascens*.

*A. thaliana* (ecotype Columbia Col-0) seeds were surface-sterilized with ethanol for 3 min followed by sodium hypochlorite for another 3 min, and rinsed three times with sterile water for 1 min each. Sterilized seeds were sown on 1/2 MS solid medium, subjected to stratification at 4 °C for 3 days, and then cultivated at 22 °C under a 16 h light/8 h dark cycle. The 15-day-old seedlings were transplanted into a soil-vermiculite mixture and cultivated under the same conditions.

### 4.2. Data Sources and Identification of the BpbZIP Gene Family

Based on the RNA-seq data on three different tissues of *B. purpurascens* (roots, stems, and leaves), as well as on heat-treated leaves (NCBI accession numbers: PRJNA987807, PRJNA1177679), the protein sequences deduced from RNA-seq data of *B. purpurascens* was used to construct a local protein database. Meanwhile, bZIP protein sequences of the *A. thaliana* were downloaded from the TAIR database. BLASTP searches were performed using TBtools, with *A. thaliana* bZIP protein sequences as the query and *B. purpurascens* protein sequences as the target, setting the E-value threshold to <10^−5^. Additionally, the “Simple HMMER” tool in TBtools was employed to search the local protein database using the bZIP-specific Pfam ID (PF00447). Results obtained from both methods were manually consolidated and filtered to remove redundant entries, yielding a preliminary set of *B. purpurascens* bZIP protein sequences. To ensure domain specificity, the candidate bZIP protein sequences were further aligned against the NCBI non-redundant (nr) database. Sequences lacking the bZIP domain were eliminated, resulting in a final set of *B. purpurascens* bZIP family genes.

The physicochemical properties of the BpbZIP family proteins, including molecular weight and isoelectric point, were analyzed using the ExPASy ProtParam tool (https://web.expasy.org/protparam/). Subcellular localization predictions of the BpbZIP proteins were carried out using the online tool Plant-mPLocServer (http://www.csbio.sjtu.edu.cn/bioinf/plant-multi/#).

### 4.3. Phylogenetic and Conserved Motif Analysis of BpbZIP Proteins

A total of 55 BpbZIP protein sequences from *B. purpurascens* and 78 bZIP gene sequences from *A. thaliana* were imported into MEGA11 software for phylogenetic analysis. Multiple sequence alignment was performed using the MUSCLE algorithm, and the resulting alignment was used to construct an unrooted phylogenetic tree based on the Maximum Likelihood (ML) method. The phylogenetic tree was subsequently refined and visualized using the online tool Chiplot (https://www.chiplot.online/).

Conserved motif analysis of the BpbZIP protein family was carried out using the MEME Suite 5.1.0 online tool. The number of motifs was set to 10, with all other parameters left at their default settings. The phylogenetic tree and conserved motifs of BpbZIP proteins were further visualized using TBtools.

### 4.4. Expression Pattern Analysis of BpbZIP Genes in Different Tissues and Under Heat Stress

RNA-seq data from *B. purpurascens* roots, stems, leaves, and heat-treated leaves were used to analyze the expression patterns of *BpbZIP* genes. Gene expression levels were quantified using RSEM (v1.3.1) (http://deweylab.biostat.wisc.edu/rsem/), and the expression values of individual genes were normalized and converted into TPM (Transcripts Per Million) units. Differential expression analysis was performed using the DESeq2 package. Genes were considered differentially expressed if they met the criteria of |log_2_(fold change)| ≥ 1 and adjusted *p*-value (Padjust) < 0.05. The TPM values of all genes were imported into TBtools to generate expression heatmaps for visualizing gene expression patterns.

### 4.5. RNA Extraction and RT-qPCR Analysis

The total RNA was extracted from the first fully expanded leaf at the top of *B. purpurascens* plants under control and heat stress conditions. RNA extraction was performed using the Ultrapure RNA Extraction Kit for plants (Cowin Biosciences, Taizhou, China). The RNA concentration and purity were measured using a NanoDrop 2000 spectrophotometer (Thermo Fisher Scientific, Waltham, MA, USA), and RNA integrity was assessed by agarose gel electrophoresis. First-strand cDNA synthesis was carried out using a two-step method with the Evo M-MLV RT Mix Kit (Accurate Biology, Beijing, China). To validate the reliability of the RNA-seq results, real-time quantitative PCR (RT-qPCR) was conducted on selected genes. The expression data were normalized using 18S rRNA as an internal reference. The RT-qPCR reactions were performed using the qTOWER3 G real-time PCR system (Analytik Jena AG, Jena, Germany). The thermal cycling conditions included an initial denaturation at 95 °C for 30 s, followed by 40 cycles of denaturation at 95 °C for 5 s and annealing/extension at 60 °C for 30 s. All primers were designed using Primer Premier 5.0 and are listed in Table S5. Relative gene expression levels were calculated using the 2^−ΔΔCt^ method.

### 4.6. Vector Construction and Plant Transformation

To construct the over-expression vector, the coding sequence of *BpbZIP37* without the stop codon was amplified by PCR using gene-specific primers (*35S-NocI-BpbZIP37*-F and *GFP-SpeI-BpbZIP37*-R) designed with homologous arms via Primer5.0 software Table S5. The amplified product was then inserted into the *pCAMBIA1300-35S-NocI-SpeI-eGFP-tNOS* vector using a seamless cloning approach. The *BpbZIP37* gene expression cassette on the recombinant plasmid was induced into *Nicotiana benthamiana* or *A. thaliana* via *Agrobacterium*-mediated genetic transformation with *A. tumefaciens* strain GV3101 [5,6].

Transgenic seedlings were screened on 1/2 MS medium supplemented with hygromycin (25 mg/L) and timentin (25 mg/L) under long-day growth conditions (16 h light at 70 µE·m^−2^·s^−1^/8 h dark) at 22 °C. A total of ten independent *BpbZIP37* over-expression (OE) lines (OE1–10) were obtained, among which OE6 and OE8, showing the highest expression levels, were selected for subsequent functional analyses.

### 4.7. Survival Rate and Root Length Assays Under Heat Stress

The 7-day-old seedlings of Wild-type (WT) and OE-6/8 transgenic *A. thaliana* were subjected to heat stress by placing the plates in a 45 °C incubator for 105 min, followed by recovery at 22 °C under light conditions for 3 days. Survival rate was evaluated based on leaf chlorosis, with the criterion that seedlings retaining at least one green true leaf were considered viable. Each group included three biological replicates and 10 plants were used per biological replicate for each line. Survival rate data were visualized as bar plots using GraphPad Prism 9, and statistical significance between groups was assessed using Student’s *t*-test (*p* < 0.05 considered significant).

To evaluate thermotolerance in root development, seedlings were exposed to graded high-temperature stress (45 °C for 60, 90, and 105 min), then returned to 22 °C under light conditions for an additional 5 days. Root morphology was recorded by photography, and primary root length was quantified using ImageJ-2 software. Each treatment was performed in three independent biological replicates. Statistical analysis of root length followed the same procedure as the survival rate assessment.

### 4.8. Detection of Reactive Oxygen Species and Soluble Protein Accumulation

The 15-day-old seedlings of WT, OE6, and OE8 were transplanted into soil and grown for an additional 3 days. After heat treatment at 45 °C for 105 min followed by recovery at 22 °C for 3 days, rosette leaves with similar size from WT, OE6, and OE8 plants were collected for reactive oxygen species (ROS) detection via nitroblue tetrazolium (NBT) and 3,3′-diaminobenzidine (DAB) staining methods [5,6]. Freshly prepared NBT and DAB staining solutions (10 mg dissolved in 10 mL phosphate buffer, respectively) were used to stain the leaves for 30 min. After staining, samples were completely decolorized with 75% ethanol and observed under a microscope. The activities of peroxidase (POD) and catalase (CAT) were measured using POD and CAT activity assay kits (Sangon Biotech, Shanghai, China) [5,6]. The contents of proline and soluble protein were determined using the previous method [5,6].

## 5. Conclusions

This study provided a comprehensive analysis of the *BpbZIP* gene family by integrating characteristic, phylogenetic, expression, and functional data, emphasizing BpbZIP37 as an important regulator of heat tolerance via ROS scavenging and antioxidant enzyme activation. Its findings not only advanced our understanding of high-altitude plant adaptation to heat stress but also provide practical tools for the conservation and sustainable utilization of *B. purpurascens* resources under global climate change. It also provides important references for stress resistance research in medicinal and crop plants.

## Data Availability

The RNA-seq data used in this study are available from the National Center for Biotechnology Information (NCBI) under the accession numbers PRJNA987807 and PRJNA1177679.

## Supplementary Materials

The following supporting information can be downloaded at: https://www.mdpi.com/article/10.3390/ijms262110262/s1.
