# Supplementary material for: Identification of bZIP Gene Family in Bergenia purpurascens and Functional Characterization of BpbZIP37 Under Heat Stress
Source: Int J Mol Sci. 2025 Oct 22;26(21):10262. doi: 10.3390/ijms262110262 (PMC12607824; doi:10.3390/ijms262110262)
Supplement: Supplementary file 1 [file ijms-26-10262-s001.zip › ijms-3871820-supplementary -R0929.pdf]

Supplemental figures

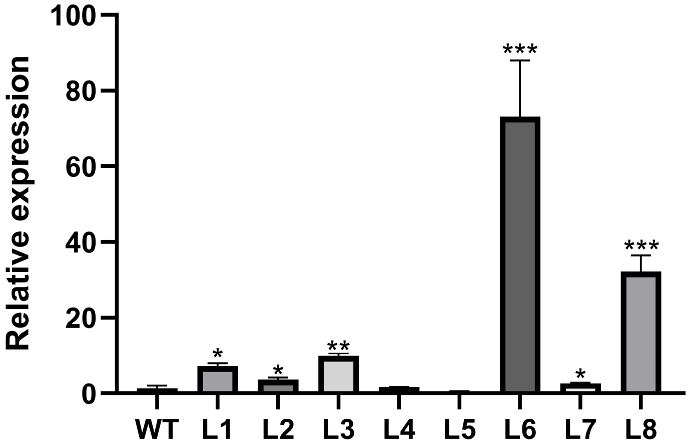

Figure S1. Relative expression levels of *BpbZIP37* gene in overexpression lines (OE1-OE8). Significance analysis was performed with WT as the control using Student's t-test (*$p < 0.05$, **$p < 0.01$, ***$p < 0.001$, n = 3)

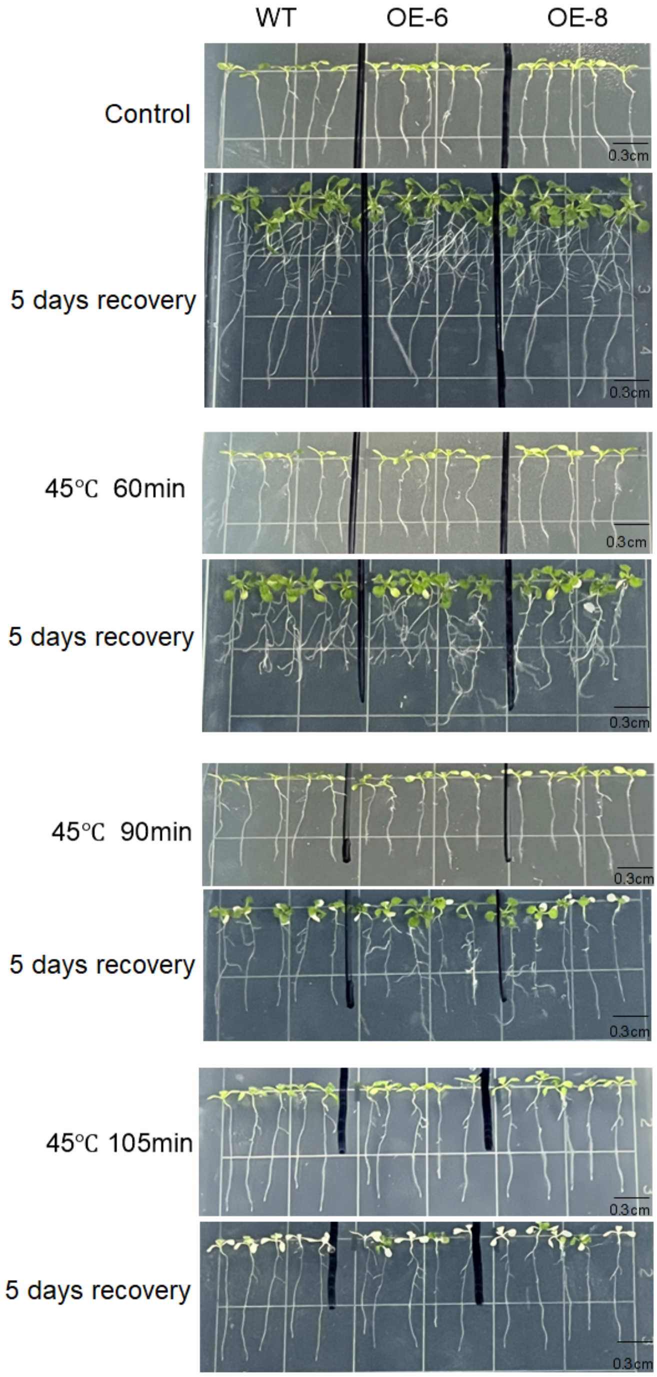

Figure S2. Phenotypes of WT and *BpbZIP37-OE* plants after heat stress. 4-day-old seedlings were treated with 45°C heat stress for different times followed by 3 days of recovery. Bar = 0.3 cm

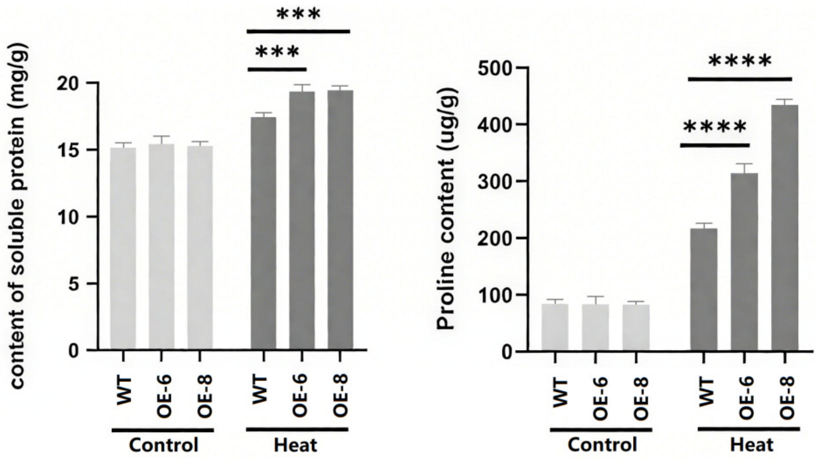

Figure S3. Soluble protein and Proline content of WT and *BpbZIP37-OE* plants after heat stress. (***$p < 0.001$, ****$p < 0.0001$, $n = 3$, Student's t-test).
